# Supplementary material for: Healthcare professionals’ awareness of the levels of disciplinary collaboration in older adults’ care during clinical practice in Nigeria: A multi-method study
Source: PLoS One. 2026 May 8;21(5):e0348887. doi: 10.1371/journal.pone.0348887 (PMC13155581; doi:10.1371/journal.pone.0348887)
Supplement: S1 File — (PDF) [file pone.0348887.s001.pdf]

Questionnaire development: face and content validity

We developed a survey instrument with standardized questions derived from a study by Okoh and colleagues [1] and from other related research papers [2–7] to purposely this study's research questions.

The questionnaire used in this study underwent both face and content validity assessments. A total of 15 healthcare professionals (HCPs), including five physicians, four physiotherapists, three nurses, two radiographers, and one social worker working in a similar setting to the target hospital, were invited to participate. The invited HCPs reviewed the questionnaire for clarity, comprehensiveness, and wording during the face validity assessment. They modified certain sections to ensure they were free from negative connotations regarding disciplinary collaborations, which they perceived as not being evident in older adult care in Nigeria. Additionally, the ordering of the questionnaire was adjusted based on their suggestions to explore the potential impact of participants' perceptions of disciplinary practice after reading the definitions of each disciplinary approach. Questions about factors influencing intra-cross or multidisciplinary gerontological practice in Nigeria were removed, as the focus was on the highest forms of disciplinary collaboration (interdisciplinary and transdisciplinary care). An additional suggestion was made to combine the questions related to barriers to interdisciplinary and transdisciplinary collaborations into a single question, asking participants to assess the extent to which these factors act as barriers to interdisciplinary/transdisciplinary collaboration in their practice center. The rationale behind this recommendation was the assumption that these factors would be similar across both collaborative practices.

For content validity, the panel of 15 HCPs rated the relevance of all questionnaire sections using a four-point ordinal scale, calculating the item-level content validity index (I-CVI), and defined the proportion of agreement on the relevancy of each item, which is between zero and one [8]. Mathematically, the I-CVI is calculated by dividing the number of experts who rated the relevancy of each item as 3 or 4 by the total number of experts [8]. According to [9], for an item to have content validity, there should be agreement higher than 79%. Additional revision is needed if the agreement falls between 70% and 79%. However, if the agreement is less than 70%, the item should be deleted from the questionnaire. We initially had 37 items, and 34 items had an agreement higher than 79%, with one item between 70 and 79% (requiring revision) and two items below 70% which were deleted. The item that requires revision was item 15; the initial question " have you heard about the definition of each term?" was revised to ask, "do you know the definition of each term?" The two deleted items were questions asking HCPs' perception (a) of disciplinary collaborations because the definition of each concept is not clear and could depend on how the HCPs understood each concept and (b) older adults/family members desire for each of these concepts. Reliability testing, specifically test-retest reliability, was not conducted in this study, because one of our study objectives, was to check if rating of the discplinary collaboration practiced in the hospital would change after reviewing the pictorial presentation as well as the written description of the dispciplinary collobartion.

After the final revision, the 35-item questionnaire was pilotested with same HCPs to determine the mintues taken to complete the survey and to understand on the challenges of using of google survey. The average time to complete the questionnaire reported by the participants was 20 mintues, with the range of 15-30 mintues. They also highlighted the challenges to using google

survey is purely on the issues related to internet, as one cannot save and continue in the google survey later.

References

1. Okoh AE, Akinrolie O, Bell-Gam HI, Adandom I, Ibekaku MC, Kalu ME. Nigerian healthcare workers' perception of transdisciplinary approach to older adults' care: A qualitative case study. Int J Care Coord. 2020;23: 92–106. doi:10.1177/2053434520954362

2. Choi BCK, Pak AWP. Multidisciplinarity, interdisciplinarity and transdisciplinarity in health research, services, education and policy: 1. Definitions, objectives, and evidence of effectiveness. Clinical and Investigative Medicine. 2006;29: 351–364.

3. Brazile T, Hostetter Shoop G, McDonough CM, Van Citters DW. Promoting innovation: Enhancing transdisciplinary opportunities for medical and engineering students. Medical Teacher. 2018. pp. 1264–1274. doi:10.1080/0142159X.2018.1426841

4. Gocan S, Laplante MA, Woodend K. View of Interprofessional Collaboration in Ontario's Family Health Teams: A Review of the Literature. J Res Interprof Pract Educ. 2014;3.

5. Ellapen TJ, Swanepoel M, Qumbu BT, Strydom GL, Paul Y. Interprofessional knowledge and perceptions of selected South African healthcare practitioners towards each other. Afr J Health Prof Educ. 2018;10: 148. doi:10.7196/AJHPE.2018.V10I3.951

6. Chew BH, Tang CJ, Lim WS, Yap JKY, Zhou W, Liaw SY. Interprofessional bedside rounds: Nurse-physician collaboration and perceived barriers in an Asian hospital. https://doi.org/101080/1356182020191566218. 2019;33: 820–822. doi:10.1080/13561820.2019.1566218

7. Iyoke CA, Lawani LO, Ugwu GO, Ajah LO, Ezugwu EC, Onah P, et al. Knowledge and attitude toward interdisciplinary team working among obstetricians and gynecologists in teaching hospitals in South East Nigeria. J Multidiscip Healthc. 2015;8: 237–244. doi:10.2147/JMDH.S82969

8. Lynn MR. Determination and quantification of content validity. Nursing Research. 1986;35: 382–386. doi:10.1097/00006199-198611000-00017

9. Abdollahpour E, Nejat S, Nourozian M, Majdzadeh R. The process of content validity in instrument development. Iranian Epidemiology. 2010;6: 66–74.
