# Supplementary material for: Healthcare professionals’ awareness of the levels of disciplinary collaboration in older adults’ care during clinical practice in Nigeria: A multi-method study
Source: PLoS One. 2026 May 8;21(5):e0348887. doi: 10.1371/journal.pone.0348887 (PMC13155581; doi:10.1371/journal.pone.0348887)
Supplement: Attachment — Submitted filename: PONE-D-24-38311_Response to Reviewers.docx [file pone.0348887.s003.docx]

**Manuscript ID: PONE-D-24-38311**
Response to Reviewers

**ACADEMIC EDITOR:**

Overall assessment:

The manuscript addresses an important issue concerning interprofessional collaboration in geriatric healthcare in Nigeria. The study's focus on disciplinary collaboration levels and barriers provides valuable insights for strengthening geriatric healthcare delivery. However, revisions are required to improve clarity, methodological transparency, and consistency in reporting. Strengthening the discussion on policy implications and refining the statistical presentation will enhance the manuscript’s contribution to the field.

We appreciate your favourable review of our manuscript. We also appreciate the time and energy you have taken to provide us with constructive comments for improvement.

Major comments:

1. The manuscript discusses various levels of disciplinary collaboration (intradisciplinary, multidisciplinary, interdisciplinary, etc.), but the operational definitions and distinctions between these concepts need to be more explicitly clarified. Ensure that the terms are consistently applied throughout.

Thank you for your comment. Figure 1 offers additional clarity, delineating increasing level of close interaction between healthcare professionals from different professions, especially from multidisciplinary to transdisciplinary collaboration. We have also ensured that these terms are consistently applied throughout the paper.

2. The study employs a cross-sectional design, but further justification is needed regarding the sampling approach. Was convenience sampling the most appropriate method? How did the authors address potential biases in the participant selection process?

Thank you for highlighting the mistake. We actually used a criterion-based purposive sampling technique. Regarding how we managed potential biases:

- Primarily, we sent out emails to a few potential participants who email addresses we obtained through our professional networks. ***Self-selection bias and gatekeeper bias*** could skew the sample toward overrepresentation of participants with certain desirable attributes who may differ systematically from the target population. To control for this influence, we engaged in direct/in-person recruitment visits to the hospital and met several clinical staff (potential participants). We also circulated the study advert on the staff WhatsApp groups and information boards at the hospital premises.
- We were mindful of **sampling homogeneity**; hence, we ensured that we achieved a diverse sample comprising doctors, physiotherapists, nurses, etc.
- We controlled **confirmation bias** as per how we defined the criteria or avoided seeking ideal participant who may confirm our hypothesis. To address this, we ensured that our sample was not too narrow but diverse and defined based on the existing literature (For example – Okoh AE, Akinrolie O, Bell-Gam HI, Adandom I, Ibekaku MC, Kalu ME. Nigerian healthcare workers’ perception of transdisciplinary approach to older adults’ care: A qualitative case study. Int J Care Coord. 2020; 205343452095436. doi:10.1177/2053434520954362)

3. The questionnaire development process should be elaborated. Was it pilot-tested or validated before use? If yes, provide details on how its reliability and validity were assessed.

We did not find an established, standardized questionnaire to adapt for this study. Thus, we developed one based on questions elicited from reviewing the literature on disciplinary collaboration in healthcare. We did not include it in the main manuscript because of word limits. We provided a detailed description of the face and content validation process in the supplementary file. We also referred readers to the supplementary file (see ‘Questionnaire structure’, page 10).

4. The results section presents descriptive and inferential statistics, but further explanation is needed regarding why specific tests (e.g., chi-square, logistic regression, Wilcoxon signed-rank test) were chosen for different analyses.

We are unable to include every detail in the manuscript because of word limits. Justifications for the statistical tests, include:

- Chi-square test is suitable for determining if there was a significant association between categorical variables.
- Logistical regression to predict their awareness of inter/transdisciplinary because we had a binary dependent variable (awareness [yes or no]). Also, other assumptions were met including independence of observations, no multicollinearity, and our sample size was large enough with respect to the number of variables.
- Freidman test is the nonparametric alternative to repeated measure ANOVA. We used it because mainly because we had five groups (i.e., more than two groups), and we treated the levels of disciplinary collaboration as ordinal variables. We also had three measurements timepoints: baseline measure followed by two post measures after pictorial and written descriptions of the disciplinary collaboration constructs.
- Wilcoxon Wilcoxon signed-rank test was used for post hoc analysis to identify where the significant difference lied from the baseline to the post measures. We used Wilcoxon signed-rank test (the nonparametric alternative to the paired t-test) because of the ordinal variables.

5. In Table 3 (regression model), provide odds ratios with confidence intervals to enhance interpretability.

We have provided odds ratios with confidence intervals as advised (see page 13)

6. The discussion should better connect the findings to practical implications for healthcare policy and practice in Nigeria. How do the barriers identified align with existing healthcare system challenges?

We have revised the discussion, connected our findings to the literature and the broader Nigerian policy and practice context.

7. While recommendations for training and policy change are mentioned, more concrete suggestions on how interdisciplinary collaboration can be integrated into healthcare education and practice would strengthen the discussion.

We have integrated this suggestion in the discussion. We highlighted transdisciplinary practice curriculum content and transdisciplinary geriatric care practice standards. We also proposed using improved government investments and targeted incentives for transdisciplinary practice to enhance uptake.

Minor comments:

1. The manuscript contains some grammatical errors and typographical issues (e.g., “transdiscplinary” should be “transdisciplinary” in the conclusion section of the abstract). A thorough proofreading is recommended.

Thank you for highlighting these grammatical and typographical errors. We have corrected them many more throughout the paper.

2. The abstract should explicitly state key numerical findings (e.g., awareness percentages) to provide a clearer summary of results.

We have provided the numbers for the results (see Abstract, page 3)

3. Ensure adherence to the PLOS ONE data availability policy—the statement should clarify how other researchers can access the dataset.

We have indicated that the dataset is available upon request by contacting the correspond author.

**Review Comments to the Author**

Please use the space provided to explain your answers to the questions above. You may also include additional comments for the author, including concerns about dual publication, research ethics, or publication ethics. (Please upload your review as an attachment if it exceeds 20,000 characters)

Reviewer #1: 1. Is the manuscript technically sound, and do the data support the conclusions?

The study "Healthcare professionals’ perceptions of the disciplinary collaborations in older adults’ care during clinical practice in Nigeria: A cross-sectional study" is relevant both in terms of the specific healthcare needs of older adults and in the broader context of improving healthcare systems in low-resource settings like Nigeria. It has the potential to contribute to knowledge, however, not all the data are provided.

Thank you for your positive comments. We have added the tables that were missing initially. The dataset generated and analysed during this study would not be publicly available because individuals’ privacy could be compromised, but it would be available from the corresponding author upon reasonable request, while ensuring ethical guidelines are adhered to.

2. Has the statistical analysis been performed appropriately and rigorously?

I don't know.

The discussion statistical analysis is good, but I cannot confirm because there are no tables. Also, they did not explain why they chose convenience sampling over probability sampling. They should explain that and justify their choice. Same applies to the sample units. Why did they settle on those they chose and not any other? These should be provided.
We have provided the tables as recommended. We chose a nonprobability sampling technique over probability sampling technique because we did not have direct access to the full contact list of care providers in the study setting, including their emails and occupation. We had a few email addresses (less than 15% of the total sample) through our professional networks. We relied on some staff to circulate the study advert in their WhatsApp groups. We posted adverts on notice boards and also went on site visits for some in-person recruitment efforts. We used criterion sampling as we have already described above because we sought to elicit diverse and representative data that offers rich insight from different disciplines. We believe that collaborative practice would be better studied by seeking insights from a sample that comprises diverse healthcare professional groups.

3. Have the authors made all data underlying the findings in their manuscript fully available?

No, the authors did not provide all available data. Even though there are indications of where tables and figures should be, no tables are found at the tail end of the document, nor are they found among the support documents. Only a figure is provided at the end of the document, and the only support document provided is the questionnaire.
We have provided the tables at the tail end of the manuscript.

4. Is the manuscript presented in an intelligible fashion and written in standard English?

Yes.
Largely the fashion and standard of English is good except for some few typos. E.g., these sentences 1. In paragraph 1 under discussion, the last two sentences "This highlighted the need to provide a concensus-contextual definitions of each concept to enhance collaborative that acualises the cultural distinctions across each practice settings" and "Moreso, it is plausible that a detailed description of these disciplinary collaboration, placed in clinics is a starting point to enhancing HCPs’ understanding of these concepts" are difficult to understand. Same apples to the second sentence in paragraph 2. e.g., "A considerable number of our study participants reported that they were awareness of the collaborative practice models, especially, intradisciplinary, multidisciplinary, and interdisciplinary collaboration." does not read well.
Thank you for highlighting these grammatical and typographical errors. We have corrected them many more throughout the paper.

These sentences should be rephrased.

Reviewer #2: The manuscript by Augustine Chukwuebuka Okoh and co-workers describes a cross-sectional survey among Nigerian Healthcare professionals to assess their perception of disciplinary collaborations in older adult care. The language of the manuscript is clear and concise with well-explained methodology allowing the reader to appreciate the study and findings. I find the objectives and study design well-aligned and statistical tools appropriately applied to report suitable findings. Although part of a larger study, nevertheless the findings here contribute to the paucity of information on disciplinary collaboration in geriatric practice in Nigeria, by adding to an understanding of the barriers and challenges to adopting this approach to the care of older adults.

Thank you for your favourable review of our manuscript.
