# Supplementary material for: Healthcare professionals’ awareness of the levels of disciplinary collaboration in older adults’ care during clinical practice in Nigeria: A multi-method study
Source: PLoS One. 2026 May 8;21(5):e0348887. doi: 10.1371/journal.pone.0348887 (PMC13155581; doi:10.1371/journal.pone.0348887)
Supplement: Attachment — Submitted filename: PONE-D-24-38311R1_Response to Reviewers.docx [file pone.0348887.s004.docx]

**Manuscript ID: PONE-D-24-38311R1**
Response to Reviewers

**Additional Editor Comments:**

Thank you for submitting the revision earlier. While reviewer showed positive response to your revised draft, he still has some concerns for which I again invite you to go through those comments and revise the draft accordingly.

We appreciate your kind words and appreciate the time and energy you have taken to provide us with constructive feedback. We have made concerted efforts to address Reviewer #1’s concerns.

Please get your draft proofread from a native English speaker and duly acknowledge that person within your draft.

We have had a native speaker (who was born and has lived all their life in Canada) proofread the manuscript, and they have been acknowledged.

**Review Comments to the Author**

Reviewer #1: Overall assessment: The study topic “Healthcare professionals’ perceptions of the disciplinary collaborations in older adults’ care during clinical practice in Nigeria” is interesting and highly relevant. If shaped well, the study stands a chance of contributing significantly to knowledge in the healthcare sector, particularly in enhancing healthcare provision for older adults. The contents are presented with a reasonable level of structure and detail, except for some few issues that limit its rigor and clarity. Below are my comments:
1. The topic implies the study seeks to establish “Healthcare professionals’ perceptions of the disciplinary collaborations in older adults’care during clinical practice in Nigeria. Measurement of perception is different from measurement of awareness. I have a challenge understanding how perception can be taken as awareness.

Thank you for your comment. We actually examined participants’ awareness level. We have replaced mentions of ‘perception’ in the manuscript with ‘awareness’.

2. The rationale for choosing a cross-sectional design is not clearly stated. Given the goals (e.g., assessing perceptions and testing influence of interventions like pictorial/written descriptions), a mixed-method or longitudinal design could potentially provide more depth so, if you used cross-sectional design, you need to justify the use of a cross-sectional design.

Thank you for point this out. After due consideration, we believe that a multi-method study would be a more accurate description of the study approach. It comprises both cross-sectional and pretest-posttest study design, with the pre-post component testing the influence of the intervention.

3. The calculation of the sample size is not clear. Though they used a formula for an unknown population, the study should clearly show how the calculation was done. For instance, the assumptions such as expected proportions are not stated.
Thank you for your comment. We have added the formular and outline parameter as follows:

“The sample size (n) was calculated using the sample size determination formula for an unknown population as described by Naing et al.

n = *Z^2^* *P(*1 – *P)*

*d^2^*

We assumed a *Z* statistic for a level of confidence (*Z*) of 95% (95% CI corresponds to *Z* value of 1.96), a degree of precision (*d*) of 5%, and expected prevalence or proportion (*P*) as 80%, thus, the study estimated sample size was 245.”

4. There is a difference between convenience sampling and purposive sampling. With purposive sampling you choose those with key information. But the process described with flyers distributed for people who “ self-identified as licensed professional in their respective profession at the time of this study, as no records were publicly available to verify their licensure” and who are willing to participate, indicates it is convenience sampling. Purposive sampling chooses key informants that the researchers know and are aware of.
Thank you for the correction. We have reframed the sampling technique as convenience sampling.

5. Some of the issues raised by reviewers have not been addressed. E.g.,

a. About pilot testing the questionnaire, this has also not been addressed.

While your question was about pilot testing, the questionnaire, we can only report what we did – face and content validity. A detailed description of the face and content validation process can be found in the supplementary file. We also referred readers to the supplementary file (see ‘Questionnaire structure’, page 10).

b. Relating to the results section where further explanation is needed and explanation for specific tests such as chi-square, logistic regression, Wilcoxon signed-rank test) were chosen for different analyses.

Justifications for the statistical tests, include:

- Chi-square test is suitable for determining if there was a significant association between categorical variables.
- Logistical regression to predict their awareness of inter/transdisciplinary because we had a binary dependent variable (awareness [yes or no]). Also, other assumptions were met including independence of observations, no multicollinearity, and our sample size was large enough considering the number of independent variables in the model (6 variables).
- Friedman test is the nonparametric alternative to repeated measure ANOVA. We used it because mainly because we had five groups (i.e., more than two groups), and we treated the levels of disciplinary collaboration as ordinal variables. We also had three measurements timepoints: baseline measure followed by two post measures after pictorial and written descriptions of the disciplinary collaboration constructs.
- Wilcoxon Wilcoxon signed-rank test was used for post hoc analysis to identify where the significant difference lied from the baseline to the post measures. We used Wilcoxon signed-rank test (the nonparametric alternative to the paired t-test) because of the ordinal variables.

These have also not been addressed with a lame explanation of word count limit.

The rationale for using each statistical test has been concisely described in the data analysis sub-section.

6. Clarity of language: The paper needs to be proofread. For instance the opening statement of the introduction does not read well. E.g. “Globally, the population of older adults is increasing rapidly, by 2050, and the number of people aged 60 years and over is projected to outnumber children aged 0-14 [1].”

Thank you for the comment. A native English language speaker has proofread the manuscript thoroughly.

Reviewer #3: Thank you for inviting me to review this manuscript on Healthcare professionals’ perceptions of the disciplinary collaborations in older adults’ care during clinical practice in Nigeria: A cross-sectional study. While this work provides a significant information about the perception of healthcare professionals about collaboration to support older adults in Nigeria, it further amplifies the voices of healthcare professionals whose voices remain invisible in research.

Thank you for your favourable review of our manuscript.

Reviewer #2: The manuscript by Augustine Chukwuebuka Okoh and co-workers describes a cross-sectional survey among Nigerian Healthcare professionals to assess their perception of disciplinary collaborations in older adult care. The language of the manuscript is clear and concise with well-explained methodology allowing the reader to appreciate the study and findings. I find the objectives and study design well-aligned and statistical tools appropriately applied to report suitable findings. Although part of a larger study, nevertheless the findings here contribute to the paucity of information on disciplinary collaboration in geriatric practice in Nigeria, by adding to an understanding of the barriers and challenges to adopting this approach to the care of older adults.

Thank you for your favourable review of our manuscript.
