# Supplementary material for: Healthcare professionals’ awareness of the levels of disciplinary collaboration in older adults’ care during clinical practice in Nigeria: A multi-method study
Source: PLoS One. 2026 May 8;21(5):e0348887. doi: 10.1371/journal.pone.0348887 (PMC13155581; doi:10.1371/journal.pone.0348887)
Supplement: Attachment — Submitted filename: PONE-D-24-38311R2_Response to Reviewers.docx [file pone.0348887.s005.docx]

**Manuscript ID: PONE-D-24-38311R2**
Response to Reviewers

**Editor Comments:**

… we invite you to submit a revised version of the manuscript that addresses the points raised during the review process.

We appreciate the time and energy you have dedicated to this manuscript’s review. We have cleaned the references, and no further changes were requested.

*Journal Requirements:*

If the reviewer comments include a recommendation to cite specific previously published works, please review and evaluate these publications to determine whether they are relevant and should be cited. There is no requirement to cite these works unless the editor has indicated otherwise.

No new references were suggested by the reviewer.

Please review your reference list to ensure that it is complete and correct. If you have cited papers that have been retracted, please include the rationale for doing so in the manuscript text, or remove these references and replace them with relevant current references. Any changes to the reference list should be mentioned in the rebuttal letter that accompanies your revised manuscript. If you need to cite a retracted article, indicate the article’s retracted status in the References list and also include a citation and full reference for the retraction notice.

The reference list has been cleaned and duplicates remove.

Also, the following references are no longer available online:

- HelpAge. Country ageing data | Data | Global AgeWatch Index 2014. 2019 [cited 15 Aug 2020] pp. 1–4. Available: https://www.helpage.org/global-agewatch/population-ageing-data/countryageing-data/?country=South%2BAfrica

They have been replaced with up-to-date sources:

- Navaneetham K, Arunachalam D. Global Population Aging, 1950-2050. Handbook of Aging, Health and Public Policy: Perspectives from Asia. 2025; 99–116. doi:10.1007/978-981-99-7842-7_154

The following bibliographic details have been updated:

- *United Nations Department of Economic and Social Affairs. World Population Ageing 2019. World population ageing 2019. 2019.*

As seen below:

- United Nations Department of Economic and Social Affairs. World Population Ageing 2019. World population ageing 2019. 2019. Available: https://digitallibrary.un.org/record/3846855

**Reviewers’ Comments:**

1. If the authors have adequately addressed your comments raised in a previous round of review and you feel that this manuscript is now acceptable for publication, you may indicate that here to bypass the “Comments to the Author” section, enter your conflict of interest statement in the “Confidential to Editor” section, and submit your "Accept" recommendation.

Reviewer #3: All comments have been addressed

Thank you for your favourable review of our manuscript.

2. Is the manuscript technically sound, and do the data support the conclusions?
The manuscript must describe a technically sound piece of scientific research with data that supports the conclusions. Experiments must have been conducted rigorously, with appropriate controls, replication, and sample sizes. The conclusions must be drawn appropriately based on the data presented.

Reviewer #3: Yes

Thank you for your favourable review of our manuscript.

3. Has the statistical analysis been performed appropriately and rigorously?

Reviewer #3: Yes

Thank you for your favourable review of our manuscript.

4. Have the authors made all data underlying the findings in their manuscript fully available?
The [PLOS Data policy](http://track.editorialmanager.com/CL0/http:%2F%2Fwww.plosone.org%2Fstatic%2Fpolicies.action%23sharing/1/010f019cb96de6cd-19013089-c3f7-47da-9ccd-58acf9fbd8af-000000/RdZ7PDoGNH1WyImsIXBUskr3DQzOEk8ef1wqcqBrxbQ=253) requires authors to make all data underlying the findings described in their manuscript fully available without restriction, with rare exception (please refer to the Data Availability Statement in the manuscript PDF file). The data should be provided as part of the manuscript or its supporting information, or deposited to a public repository. For example, in addition to summary statistics, the data points behind means, medians and variance measures should be available. If there are restrictions on publicly sharing data—e.g. participant privacy or use of data from a third party—those must be specified.

Reviewer #3: Yes

Thank you for your favourable review of our manuscript.

5. Is the manuscript presented in an intelligible fashion and written in standard English?
PLOS ONE does not copyedit accepted manuscripts, so the language in submitted articles must be clear, correct, and unambiguous. Any typographical or grammatical errors should be corrected at revision, so please note any specific errors here.

Reviewer #3: Yes

Thank you for your favourable review of our manuscript.

6. Review Comments to the Author
Please use the space provided to explain your answers to the questions above. You may also include additional comments for the author, including concerns about dual publication, research ethics, or publication ethics. (Please upload your review as an attachment if it exceeds 20,000 characters)

Reviewer #3: I have gone through the revised version of the manuscript, and I have ascertained that the authors have done an amazing job. This manuscript will have significant contribution to wider readers in Africa.

Thank you for your favourable review of our manuscript.

7. PLOS authors have the option to publish the peer review history of their article ([what does this mean?](https://track.editorialmanager.com/CL0/https:%2F%2Fjournals.plos.org%2Fplosone%2Fs%2Feditorial-and-peer-review-process%23loc-peer-review-history/1/010f019cb96de6cd-19013089-c3f7-47da-9ccd-58acf9fbd8af-000000/Z6YKzi17HAk-gACjTV3_1h9mk1IcL9Kr2RdMbgRU0sk=253)). If published, this will include your full peer review and any attached files.
If you choose “no”, your identity will remain anonymous but your review may still be made public.
**Do you want your identity to be public for this peer review?** For information about this choice, including consent withdrawal, please see our [Privacy Policy](https://track.editorialmanager.com/CL0/https:%2F%2Fwww.plos.org%2Fprivacy-policy/1/010f019cb96de6cd-19013089-c3f7-47da-9ccd-58acf9fbd8af-000000/tLdRE1TbL7j3K6unOttq5jKXPEPYXbRMGKsm63Dbxow=253).

Reviewer #3: **Yes:** Oluwagbemiga Oyinlola

Thank you for your favourable review of our manuscript.
